# The CKM–GDM multi-organ precision model: reframing gestational diabetes as an early cardiovascular–kidney–metabolic event in Southeast Asia

**DOI:** 10.3389/fendo.2026.1949130

**Published:** 2026-09-29

**Authors:** Ashutosh Mishra

**Affiliations:** Panacea Multispeciality Hospital, Varanasi, India

**Keywords:** cardiovascular–kidney–metabolic, endothelial dysfunction, first trimester, gestational diabetes, precision medicine, Southeast Asia

## Abstract

**Background:**

Gestational diabetes mellitus (GDM) is amongst the commonest complications of pregnancy and an early marker of lifetime cardiovascular–kidney–metabolic (CKM) risk. Its burden is heavy in Southeast Asia, where prevalence ranges from about 8% to nearly 30% and a lean phenotype — with marked over-representation of the insulin-secretion-limited subtype — diverges from Western cohorts, yet its definition, timing, and treatment remain contested.

**Aim:**

This clinically oriented narrative review synthesises the diagnostic landscape, the shift to early diagnosis, the vascular mechanism, and precision care, centred on Southeast Asia.

**Key themes:**

Competing criteria (IADPSG one-step, WHO 2013, NICE, ADA two-step and the pragmatic Indian DIPSI test) identify different populations. The TOBOGM trial shows that early (pre-20-week) treatment improves outcomes, and first-trimester biomarkers—low adiponectin and sex-hormone-binding globulin, glycosylated fibronectin, atherogenic lipids, and multimarker or machine-learning panels including a non-fasting Asian-women panel—show potential to make early prediction feasible. Vascular and endothelial dysfunction (oxidative stress, eNOS uncoupling, sFlt-1/PlGF imbalance, and arterial stiffness) is proposed as the unifying early signal and a mechanistic bridge to later type 2 diabetes, cardiovascular disease, and chronic kidney disease. GDM is increasingly recognised as heterogeneous (insulin-resistant, insulin-deficient, and mixed subtypes), inviting subtype- and phenotype-guided precision diagnosis and treatment.

**Conclusion:**

We propose that GDM may be productively reframed as an early, vascular, and potentially precisely treatable CKM event—screened early, matched to phenotype and linked to lifelong follow-up, and fitted to the epidemiology, genetics, and health systems of Southeast Asia. This reframing is a testable hypothesis that requires prospective validation before routine adoption.

## Highlights

- What is already known. GDM affects roughly one in seven pregnancies and follows a continuous glucose–risk relationship, but several incompatible diagnostic criteria remain in use. Treating GDM reduces perinatal harm, and a history of GDM markedly raises later risk of type 2 diabetes and cardiovascular disease. GDM is pathophysiologically heterogeneous, and TOBOGM has shown that treating hyperglycaemia detected before 20 weeks improves outcomes.
- What this review adds. It integrates, for the practising clinician, the full diagnostic-criteria landscape (mmol/L and mg/dl), the emerging definition of early GDM, and first-trimester biomarker prediction. It proposes first-trimester vascular and endothelial dysfunction as the unifying early signal and the mechanism that makes GDM an early window into the CKM continuum; extends GDM subtypes into a multidimensional, multi-organ precision map with phenotype-guided treatment; and centres the Southeast Asian phenotype, epidemiology, genetics and health-system context. The models are presented as testable hypotheses.

## 1 Introduction

Gestational diabetes mellitus (GDM)—hyperglycaemia first recognised in pregnancy—affects roughly one in seven pregnancies worldwide (**Box 1**), and the burden is disproportionately Asian (1). A composite clinical vignette (**Box 1**) illustrates the problem this review addresses. In Southeast Asia, the reported prevalence spans a strikingly wide range, from about 8% in Malaysia and Myanmar to 14% in Singapore, 17% in Vietnam, and nearly 29% in Thailand, driven partly by real differences in risk and partly by the region’s lack of a common diagnostic standard (2). For decades, GDM was conceived narrowly, as a transient carbohydrate intolerance of the late second and third trimesters, screened for and treated to reduce short-term perinatal harm. That conception is being dismantled on three fronts at once. First, the diagnosis is contested: several incompatible sets of criteria remain in simultaneous use, and the field has not agreed on whom to test, when, or at what threshold. Second, the timing is shifting earlier: randomised evidence now supports treating hyperglycaemia in the first half of pregnancy, and biomarkers are making first-trimester prediction realistic. Third, the condition is being recognised as heterogeneous and lifelong—a vascular, cardiometabolic disorder that may unmask a woman’s future cardiovascular–kidney–metabolic (CKM) trajectory and that could be treated more precisely once its subtype is known (3, 4).

Box 1 **A clinical vignette (illustrative).**

 Meena, 27, is lean (body mass index 23) and lives in a North Indian town. At her first antenatal visit her fasting glucose is borderline, her adiponectin low, and her triglyceride–glucose index high; a bedside check shows early arterial stiffness. Under conventional care, she is reassured and tested only at 24–28 weeks. Eight years later, she has type 2 diabetes and early chronic kidney disease. This composite, illustrative case (**Box 1**) is the problem this review addresses: the signals that would have flagged Meena as a high-risk CKM phenotype were present, measurable, and overlooked in the first trimester.

This review integrates these threads for the practising clinician, moving from how GDM is defined, through the case for early diagnosis and the biomarkers and vascular mechanisms that enable it, to the CKM consequences that make early identification worthwhile, and finally to precision diagnosis and treatment. Its organising context is Southeast Asia, where the lean, frequently insulin-secretion-limited phenotype, the high and variable prevalence, and constrained antenatal resources make earlier and more precise care both especially valuable and especially challenging. Underlying the review is a clinical premise: the objective of GDM care is not merely to normalise glucose in pregnancy, but to anticipate and reduce long-term CKM organ-damage risk for the mother and her offspring, within the constraints of developing-country practice.

Scope and approach. This is a narrative, not a systematic, review. PubMed/MEDLINE and Google Scholar were searched to May 2026 for English-language articles combining gestational diabetes with diagnosis, early pregnancy or first trimester, biomarker or prediction, endothelial or vascular dysfunction, cardiovascular–kidney–metabolic, precision medicine or subtype, and Southeast Asia or Asian; landmark trials, consensus statements, systematic reviews and highly cited primary studies were prioritised, and reference lists were hand-searched. Selection was purposive rather than exhaustive, so the synthesis reflects the balance of current evidence rather than a formal quantitative appraisal. Importantly, the CKM–GDM models presented here—the vascular window, the multi-organ precision map, the precision-phenotyping algorithm and the clinical journey—are conceptual frameworks built from existing evidence but not yet prospectively tested; they are offered as testable hypotheses, and the research agenda in Sections 11–12 sets out how each can be validated. All cited studies have completed peer review and are fully published; where conclusions rest on the most recent (2025–2026) data, readers should note that this evidence continues to evolve.

## 2 Defining gestational diabetes: criteria and their controversies

### 2.1 From O’Sullivan to HAPO

Key terms used throughout this review are defined in **Box 2**.

Box 2 **Key definitions.**

 CKM (cardiovascular–kidney–metabolic): the interconnected syndrome linking metabolic risk factors, chronic kidney disease and cardiovascular disease, formalised by the American Heart Association (2023).

Early GDM: hyperglycaemia meeting standard (IADPSG) GDM thresholds when tested before 20 weeks, once overt diabetes in pregnancy has been excluded.

GDM phenotypes: insulin-resistant, insulin-deficient (lean) and mixed subtypes defined by insulin sensitivity and secretion—extended here by a vascular–angiogenic axis.

CKM–GDM Multi-Organ Precision Model: the framework proposed here—GDM as a multi-organ, multi-stage, multi-axis early CKM event (a conceptual, testable model).

Tier 1–3 strategy: resource-stratified assessment—Tier 1, clinical risk + glucose; Tier 2, add adipokine/lipid and bedside vascular tests; Tier 3, add advanced vascular, placental and multi-omic profiling.

GDM was first defined by O’Sullivan and Mahan in 1964 using thresholds that predicted a mother’s future diabetes rather than pregnancy outcomes (5); Carpenter and Coustan later recalibrated these for plasma glucose (6). The Hyperglycemia and Adverse Pregnancy Outcomes (HAPO) study transformed the field by demonstrating a continuous, graded relationship between maternal glucose and adverse perinatal outcomes with no clear threshold (7), forcing the field to draw diagnostic lines on a continuum.

### 2.2 The current landscape

HAPO led the International Association of Diabetes and Pregnancy Study Groups (IADPSG) to propose a one-step 75 g oral glucose tolerance test (OGTT) with a single abnormal value sufficient for diagnosis—fasting ≥5.1 mmol/L (92 mg/dl), 1h ≥10.0 mmol/L (180 mg/dl), or 2h ≥8.5 mmol/L (153 mg/dl) (8). The World Health Organization adopted these values in 2013 whilst separating “GDM” from “diabetes in pregnancy” (overt diabetes), the latter defined by fasting glucose ≥7.0 mmol/L (126 mg/dl), HbA1c ≥6.5%, or random glucose ≥11.1 mmol/L (200 mg/dl) (9). The United Kingdom’s NICE retained a two-value 75 g test emphasising fasting glucose and cost-effectiveness (10), and the American Diabetes Association endorses either the one-step IADPSG approach or a two-step 50 g screen followed by a 100 g Carpenter–Coustan OGTT (11). These criteria are not interchangeable: applied to the same women, they identify overlapping but distinct populations with different prevalence and outcome associations (**Table 1**) (12). In India and much of South Asia, the Diabetes in Pregnancy Study Group India (DIPSI) single-step, non-fasting 75 g test—a 2h venous plasma glucose ≥7.8 mmol/L (140 mg/dl) diagnosing GDM and ≥11.1 mmol/L (200 mg/dl) overt diabetes—is widely used for its field practicality, needing neither fasting nor a second visit, although it shows only moderate agreement with the IADPSG test and may under-detect fasting-predominant hyperglycaemia (46). This matters acutely in Southeast Asia, where different countries—and often different hospitals within a country—apply different criteria (2).

**Table 1:** Principal diagnostic criteria for gestational diabetes and diabetes in pregnancy, in mmol/L (mg/dl).

Criterion	Test	Glucose thresholds—mmol/L (mg/dl)	Comment
IADPSG/WHO 2013 (one-step)	75 g OGTT, ≥ 1 abnormal value	Fasting ≥ 5.1 (92); 1h ≥ 10.0 (180); 2h ≥ 8.5 (153)	HAPO-derived; higher prevalence; internationally comparable
ADA two-step (Carpenter–Coustan)	50 g GCT then 100 g OGTT, ≥ 2 values	Fasting ≥ 5.3 (95); 1 h ≥10.0 (180); 2h ≥ 8.6 (155); 3h ≥ 7.8 (140)	Traditional North American approach
NICE (UK)	75 g OGTT, ≥ 1	Fasting ≥ 5.6 (101) or 2h ≥ 7.8 (140)	Emphasises fasting glucose and cost-effectiveness
DIPSI (India)	75 g non-fasting, 2h	2h ≥ 7.8 (140) GDM; ≥ 11.1 (200) overt	Single-step, non-fasting; field-pragmatic; may miss fasting hyperglycaemia
Diabetes in pregnancy (overt)	Any	Fasting ≥ 7.0 (126); HbA1c ≥6.5%; random ≥ 11.1 (200)	Pre-existing/overt diabetes, excluded before an early-GDM label

GCT, glucose challenge test; HbA1c, glycated haemoglobin; IADPSG, International Association of Diabetes and Pregnancy Study Groups; DIPSI, Diabetes in Pregnancy Study Group India.

### 2.3 The one-step versus two-step controversy

The choice between them is genuinely unresolved. The one-step approach roughly doubles diagnosed prevalence and identifies milder hyperglycaemia, but large pragmatic trials have not shown that treating this additional group improves perinatal outcomes enough to offset the costs and medicalisation of pregnancy (13, 14). Proponents counter that the one-step method is physiologically coherent, internationally comparable, and better identifies women at future cardiometabolic risk. For a clinician, the practical consequence is that “GDM” is not one disease with one definition but a label whose meaning depends on the criteria used—a fact that becomes still more important when testing is moved into early pregnancy.

## 3 Early, first-trimester gestational diabetes

If the standard OGTT is performed at 24–28 weeks to capture the peak of placental insulin resistance, it necessarily overlooks women whose beta-cell reserve is already inadequate earlier, and it forecloses the first half of pregnancy for intervention. The Treatment of Booking Gestational Diabetes Mellitus (TOBOGM) trial addressed this directly: women with risk factors and hyperglycaemia detected before 20 weeks were randomised to immediate treatment or usual care, and early treatment reduced a composite of adverse neonatal outcomes—the first randomised demonstration that acting sooner helps (15). Early GDM is best defined as hyperglycaemia meeting standard (IADPSG-based) GDM thresholds when tested before 20 weeks, once overt diabetes in pregnancy has been excluded; in TOBOGM, such women were identified at booking and treated from early in the second trimester. Secondary analyses are refining which glucose thresholds define a clinically meaningful “early GDM” (16). Two cautions temper enthusiasm. First, early hyperglycaemia is heterogeneous: it includes undiagnosed overt diabetes, true early GDM, and women who would never meet criteria at 24–28 weeks. Second, applying late-pregnancy thresholds to early pregnancy is physiologically questionable, since glucose tolerance changes across gestation. The definition of early GDM therefore remains a work in progress—which is precisely why early prediction, rather than reliance on a single early glucose value, is attractive and why the region’s constrained antenatal contact makes a simple early test so valuable.

## 4 First-trimester prediction and biomarkers

Several classes of first-trimester marker now predict GDM beyond maternal characteristics (**Table 2**). Amongst adipokines, low circulating adiponectin is one of the most consistent early signals, substantially raising the odds of later GDM in meta-analysis (17), and low sex-hormone-binding globulin points to the same early hepatic insulin resistance. Glycosylated fibronectin, a glycoprotein of endothelial and placental origin, is elevated in the first trimester of affected women and has been proposed as a point-of-care marker (18). An atherogenic early lipid profile and a raised pregnancy-specific triglyceride–glucose index add independent value (19). Combining maternal factors with biomarkers improves discrimination: first-trimester models and targeted mass-spectrometry multimarker panels achieve areas under the receiver-operating-characteristic curve of roughly 0.75–0.80, appreciably better than clinical factors alone (20, 21), and machine-learning approaches integrating metabolomic and clinical features push performance further (22). The most consequential recent advance is a compact panel measured in a single non-fasting first-trimester sample that predicts GDM as accurately as second-trimester fasting tests; developed in an Asian-women cohort, it improved prediction beyond age, body-mass index, and family history without requiring fasting—a decisive practical advantage for routine early care in the region (23). Scoping reviews confirm both the promise and the heterogeneity of this literature and the need for standardised, comparative evaluation (24).

**Table 2:** Representative first-trimester predictors of gestational diabetes mellitus.

Predictor	Category	Direction	Comment
Fasting glucose/HbA1c	Glycaemic	↑	Basis of guideline-recommended early screening in higher-risk women
Adiponectin	Adipokine	↓	Consistent meta-analytic predictor; ethnic variation in Asian women
Sex-hormone-binding globulin	Insulin resistance	↓	Reflects early hepatic insulin resistance
Glycosylated fibronectin	Glycoprotein	↑	Proposed first-trimester point-of-care marker
Triglyceride–glucose index/lipids	Metabolic	↑	Atherogenic early profile adds independent value
sFlt-1/PlGF ratio	Angiogenic/vascular	Imbalance (↑ sFlt-1)	Links early vascular dysfunction to GDM and pre-eclampsia
Arterial stiffness/pulse-wave velocity	Vascular function	↑	Situates GDM in the cardio-renal-metabolic chain
Multimarker metabolomic/ML panels	Composite	—	AUC ≈ 0.75–0.80; non-fasting Asian-women panel matches fasting tests

AUC, area under the curve; HbA1c, glycated haemoglobin; ML, machine learning; PlGF, placental growth factor; sFlt-1, soluble fms-like tyrosine kinase-1.↑, increased or higher than reference; ↓, decreased or lower than reference. —, no consistent directional change.

These performance figures warrant caution, however. Most derive from single-centre or modest cohorts using differing assays and cutoffs; few biomarkers (adiponectin, sex-hormone-binding globulin, glycosylated fibronectin, and angiogenic ratios) have internationally standardised assays, and inter-assay and inter-laboratory reproducibility, batch effects, and the absence of agreed thresholds limit direct clinical transfer. Cost and access remain the binding constraints in low- and middle-income settings, where high-cost tests risk widening equity gaps. Because studies define GDM by different criteria (IADPSG, WHO, NICE, DIPSI, Carpenter–Coustan), cross-study comparisons of area-under-the-curve are difficult. These markers are therefore best regarded as promising but not yet clinically ready and as candidates for prospective, standardised evaluation rather than immediate adoption. Accordingly, this hypothesis and theory article treats biomarker-based early prediction as a promising research avenue rather than a recommendation for routine screening.

## 5 Vascular and endothelial dysfunction in GDM

What unites these markers is that they report on the vascular endothelium, which is dysfunctional early in GDM: hyperglycaemia and lipotoxicity reduce nitric-oxide bioavailability, impair flow-mediated dilation and degrade the endothelial glycocalyx before overt disease, coupled to an adverse adipokine shift (falling adiponectin with a rising leptin-to-adiponectin ratio) and, in parallel, a rising placental-derived sFlt-1/PlGF ratio that antagonises placental growth factor—a pattern shared with pre-eclampsia (4, 17). A combined GDM–pre-eclampsia vascular–placental panel, with point-of-care markers such as glycosylated fibronectin, could stratify higher-risk women for closer surveillance. Increased arterial stiffness and pulse-wave velocity can be demonstrated in affected pregnancies, situating GDM within the cardio-renal-metabolic chain (25). Because these changes are present early and quantifiable non-invasively, they are attractive first-trimester predictors—and, unlike a glucose value alone, they report on the mechanism of harm rather than only its presence. The molecular steps behind this are described once, as a single cascade, below (**Figure 1**); later sections cross-refer to **Figure 1** and Section 5 rather than repeating them.

**Figure 1:**
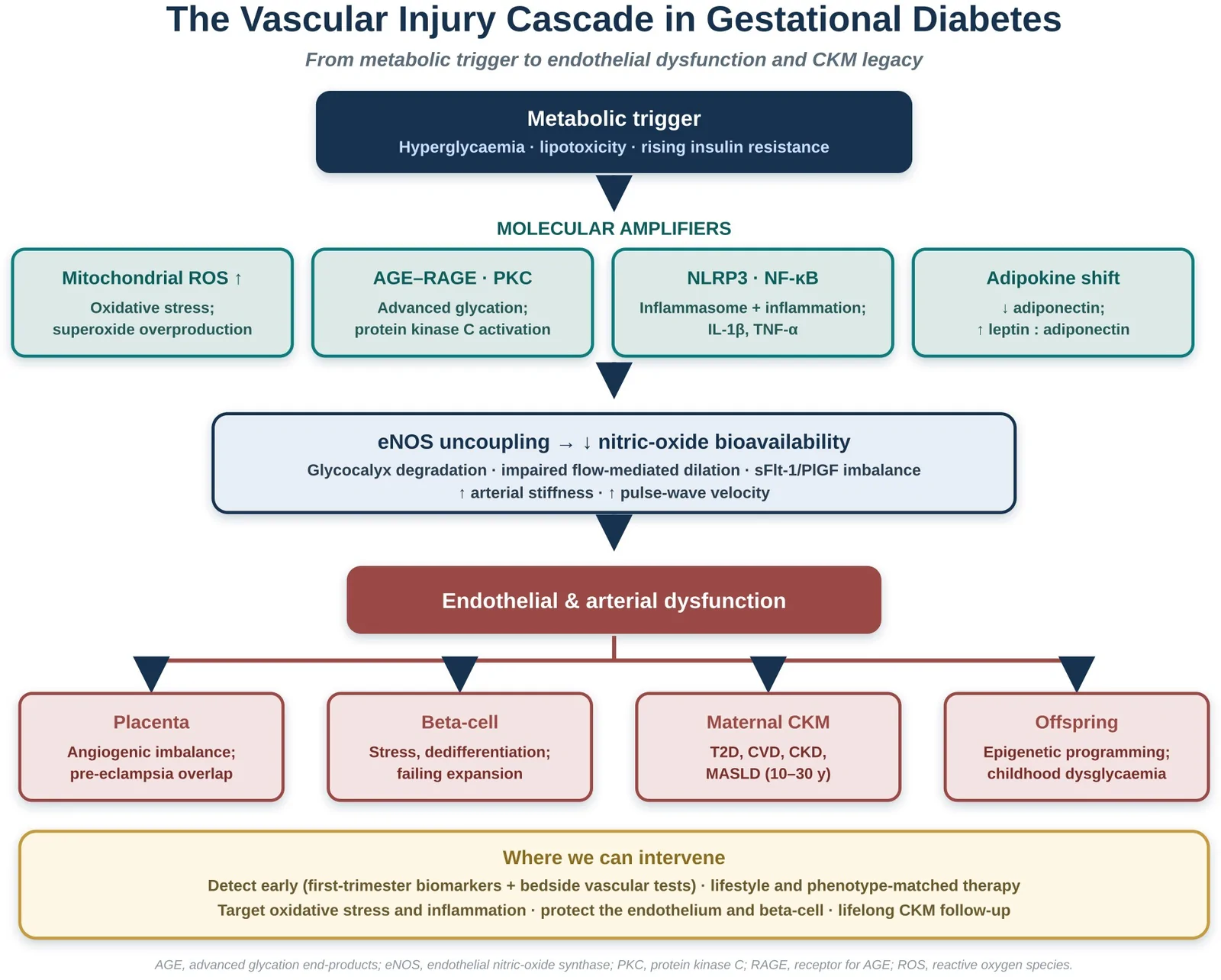
The vascular injury cascade in gestational diabetes. A metabolic trigger (hyperglycaemia, lipotoxicity, insulin resistance) activates molecular amplifiers—mitochondrial reactive-oxygen-species, advanced glycation (AGE–RAGE) and protein kinase C, NLRP3-inflammasome and nuclear factor-κB inflammation, and an adverse adipokine shift—that uncouple endothelial nitric-oxide synthase and lower nitric-oxide bioavailability, producing endothelial and arterial dysfunction. From this shared path the placental, beta-cell, maternal CKM and offspring branches diverge; each amplifier is also a point of intervention. AGE, advanced glycation end-products; eNOS, endothelial nitric-oxide synthase; PKC, protein kinase C; RAGE, receptor for AGE; ROS, reactive oxygen species.

The cascade (**Figure 1**) runs as follows. A metabolic trigger—hyperglycaemia, lipotoxicity and rising insulin resistance—activates a set of amplifiers that recur across organs (48): mitochondrial reactive-oxygen-species generation, advanced glycation (AGE–RAGE) and protein kinase C signalling, and NLRP3-inflammasome and nuclear factor-κB inflammation, together with the adverse adipokine shift. Converging on the endothelium, these uncouple endothelial nitric-oxide synthase, lower nitric-oxide bioavailability and raise arterial stiffness—the shared final common path from which the placental, beta-cell, maternal CKM and offspring branches diverge. Framing the mechanism this way shows where care can act: detect the cascade early and target oxidative stress, inflammation and endothelial protection rather than glucose alone.

### 5.1 Placenta-specific biomarkers and clinical stratification

Several of the most useful early signals are placental in origin, giving them both mechanistic meaning and practical appeal. Glycosylated fibronectin, released from placental and endothelial tissue, rises in the first trimester and suits a single-sample, point-of-care lateral-flow format deployable at a first antenatal visit without fasting or a laboratory (18). The sFlt-1/PlGF ratio links GDM biologically to pre-eclampsia; a combined vascular–placental panel could triage women who share this endothelial–placental phenotype into closer antenatal surveillance, earlier postpartum CKM follow-up, and coordinated obstetric–diabetes care (4). Beyond these markers, placenta-derived microRNAs, extracellular vesicles, and proteomic and metabolomic signatures are emerging as richer readouts of placental stress; for now they are research tools appropriate to tertiary “Tier 3” settings, but they are the most likely source of the next generation of early, mechanism-based tests (24). The clinical logic is consistent: use whatever placental marker a centre can afford to identify the high-risk vascular–placental phenotype and let an abnormal result intensify surveillance rather than sit unused.

## 6 Gestational diabetes as a window into the CKM continuum

Vascular and metabolic abnormalities associated with GDM may persist beyond delivery in many women. Women with a history of GDM have a roughly 10-fold higher risk of progressing to type 2 diabetes than parous women without GDM (26) and an elevated long-term risk of cardiovascular disease only partly explained by intervening diabetes (27, 28). The kidney is increasingly implicated, with GDM associated with later albuminuria and chronic kidney disease (29). GDM also tracks with later metabolic dysfunction-associated steatotic liver disease (MASLD) and subclinical cardiac changes such as left-ventricular diastolic dysfunction, rounding out the full CKM phenotype (47). Persistent endothelial dysfunction and adverse adipokine and inflammatory signatures provide a coherent mechanistic bridge between the index pregnancy and this later CKM disease (4). The exposure is intergenerational: offspring of GDM pregnancies carry increased risks of obesity and dysglycaemia (30), consistent with the developmental origins of health and disease, in which the intrauterine metabolic environment may programme long-term offspring risk through epigenetic mechanisms (49). Framed this way, GDM may be hypothesised to be an early, sex-specific manifestation of the CKM continuum—a vascular stress test that identifies women whose lifetime trajectory is already diverging and in whom early identification could create a rare opportunity for primordial prevention. In Southeast Asia, where cardiometabolic disease emerges early and at low body-mass thresholds, this window may open sooner and matter more.

These observations can be drawn together into a single organising framework, which we term the CKM–GDM Vascular Window Model (**Figure 2**). In this model the lean, insulin-resistant, or insulin-deficient South and Southeast Asian phenotype, with its characteristic susceptibility loci, is the substrate on which early hyperglycaemia acts; first-trimester metabolic and angiogenic signals mark the transition; endothelial and arterial injury is the proposed shared mechanism; and progression to maternal type 2 diabetes, and cardiovascular and kidney disease—together with offspring programming—is the outcome. The value of the model, if validated, is that the vascular injury may be detectable and modifiable in a first-trimester “window” that opens before conventional CKM markers become abnormal, giving a resource-stratified developing-country pathway a concrete target for early, mechanism-based prevention.

**Figure 2:**
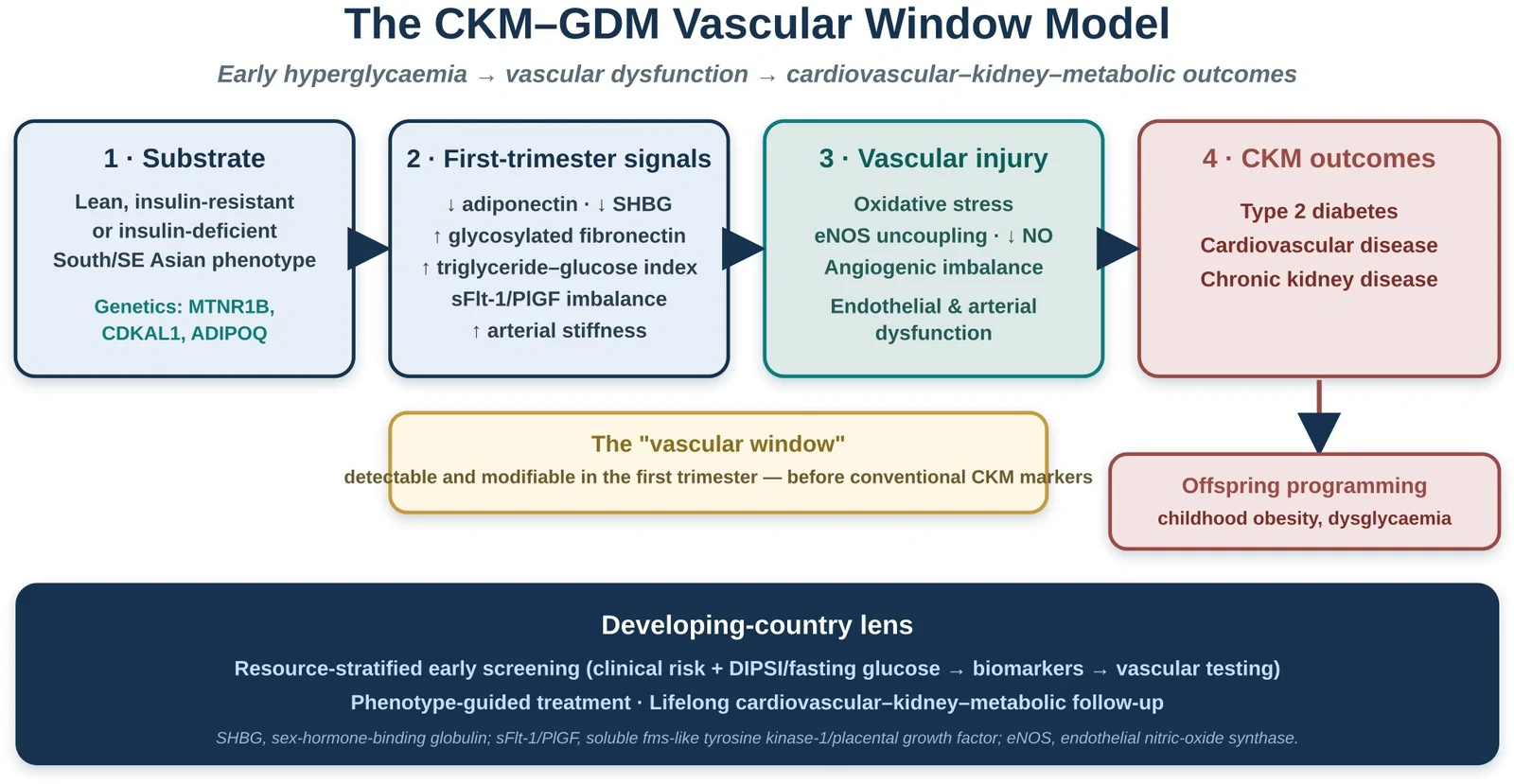
The CKM–GDM vascular window model. Early hyperglycaemia in a susceptible South/Southeast Asian phenotype produces first-trimester metabolic and angiogenic signals and endothelial injury that may precede—and drive—the maternal CKM continuum and offspring programming. The first-trimester “vascular window” is hypothesised to be detectable and modifiable before conventional CKM markers, and can be approached through a resource-stratified developing-country pathway. Conceptual, hypothesis-generating schematic integrating existing evidence; not yet prospectively validated.

Complementing this trajectory model, the woman who traverses it can be described by a CKM–GDM vascular phenotype: a proposed early, sex-specific expression of the CKM syndrome formalised by the American Heart Association (47), characterised not by hyperglycaemia alone but by a distinctive pattern of endothelial injury, reduced nitric-oxide bioavailability, angiogenic (sFlt-1/PlGF) imbalance and increased arterial stiffness measurable in the first trimester. Defining GDM this way—inputs (first-trimester glycaemia, adipokines, angiogenic markers, arterial stiffness), an intermediate vascular phenotype, and outputs (maternal 10–30-year CKM events and intergenerational metabolic risk)—reframes it from a transient obstetric problem into a candidate entry point of the CKM continuum, and gives clinicians and researchers a single labelled construct to test and to act on, though one that remains a hypothesis until validated in outcome studies. Because the pathways that injure the endothelium in GDM are the same processes that drive established CKM disease, strategies targeting oxidative stress, inflammation and endothelial function could in principle carry a dual benefit—a hypothesis that requires direct testing.

### 6.1 The CKM–GDM multi-organ precision model

We propose—as the central, testable hypothesis of this article—that GDM may be read as an early, multi-organ CKM event, captured by the CKM–GDM Multi-Organ Precision Model (**Figure 3**). The model is deliberately three-dimensional. It is multi-organ, spanning skeletal muscle and liver, the placenta and endothelium, the pancreatic beta cell, and—as disease evolves—the kidney and heart. It is multi-stage, tracing the same woman from a pre-pregnancy metabolic substrate through early and late pregnancy to the postpartum years and into her offspring. And it is multi-axis, describing each woman by her glycaemic, vascular-angiogenic, beta-cell and cumulative CKM-risk status rather than by a single glucose threshold. Read together, the concentric structure places the GDM episode at the centre, the metabolic and placental–vascular mechanisms in the surrounding rings, and the long-term CKM outcomes at the periphery. It is a conceptual model intended to generate testable predictions rather than an established classification; the associations it integrates should be read as hypothesis-generating until prospectively validated (47).

**Figure 3:**
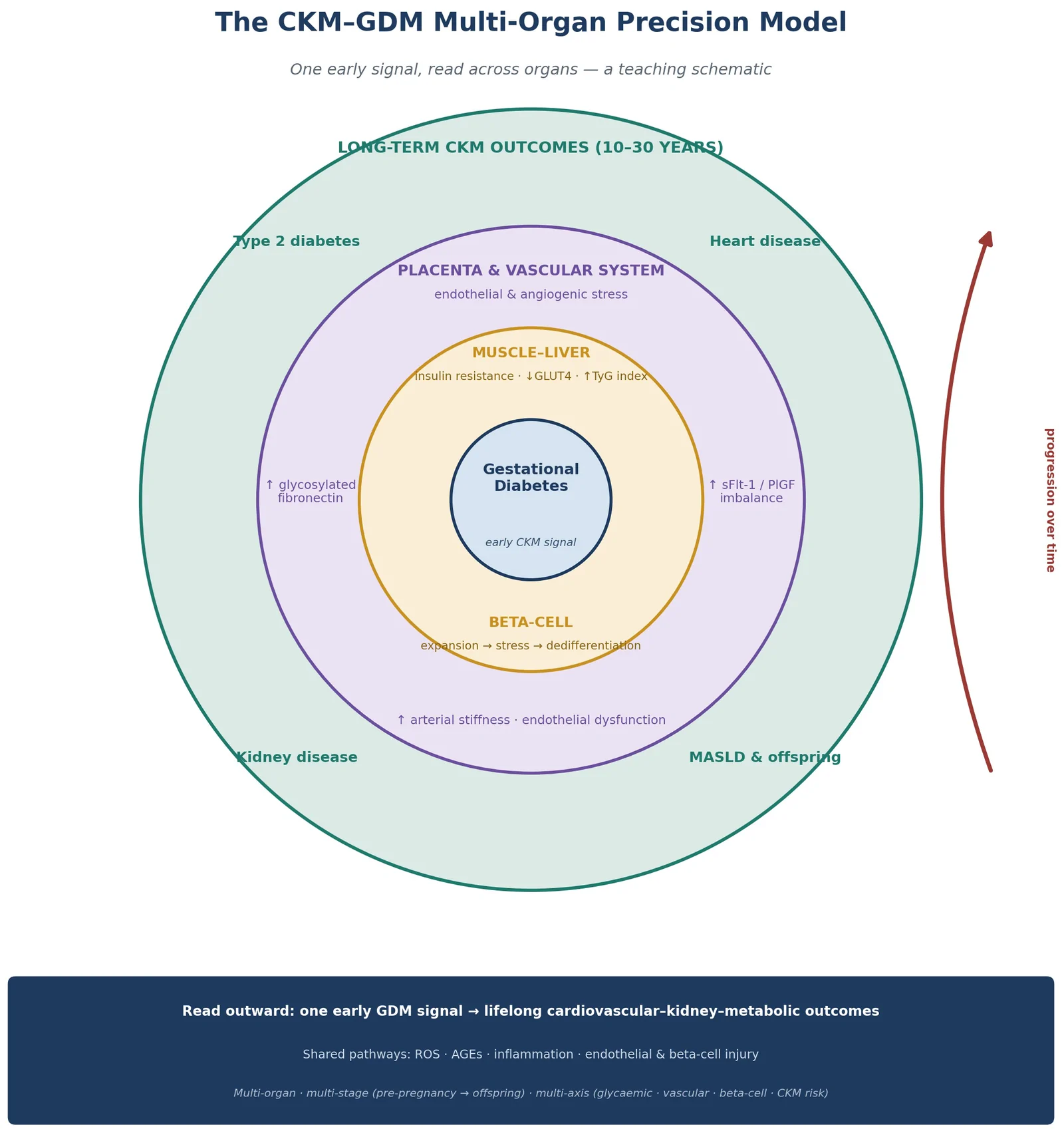
The CKM–GDM multi-organ precision model (teaching schematic). The gestational diabetes episode (centre) sits within a metabolic core (muscle–liver insulin resistance; beta-cell expansion, stress and dedifferentiation), a placental–vascular interface (glycosylated fibronectin, sFlt-1/PlGF imbalance, arterial stiffness and endothelial dysfunction), and an outer ring of long-term CKM outcomes over 10–30 years—type 2 diabetes, heart disease, kidney disease, MASLD and offspring metabolic risk. Read outward, one early GDM signal is proposed to foreshadow lifelong CKM disease through shared pathways (reactive-oxygen-species, advanced glycation, inflammation, endothelial and beta-cell injury). The model is deliberately multi-organ, multi-stage and multi-axis. Conceptual, hypothesis-generating schematic integrating existing evidence; not yet prospectively validated.

### 6.2 Stage-wise, multi-organ involvement

The model becomes clinically useful when read stage by stage and organ by organ (**Table 3**). Before and in early pregnancy, skeletal muscle and liver carry a subclinical insulin-resistance burden — reduced GLUT4-mediated glucose disposal, a raised triglyceride–glucose index and low adiponectin — that is often silent but sets the stage on which placental insulin resistance later acts. As pregnancy advances, the placenta and endothelium express overt stress through glycosylated fibronectin, the sFlt-1/PlGF shift and glycocalyx injury, whilst the beta cell, having expanded to meet rising demand, may begin to fail. The kidney shows hyperfiltration and, in some women, early albuminuria; the heart and vasculature register increased arterial stiffness and reduced nitric-oxide bioavailability. After delivery, none of these changes fully resolves: persistent muscle insulin resistance, reduced beta-cell reserve, vascular “memory” and early kidney strain together drive the 10–30-year risk of type 2 diabetes, cardiovascular disease, chronic kidney disease and MASLD.

**Table 3:** Stage-wise, multi-organ involvement in the CKM–GDM multi-organ precision model.

Organ/tissue	Pre-/early pregnancy	Late pregnancy	Postpartum and CKM legacy
Skeletal muscle & liver	Subclinical insulin resistance; ↓ GLUT4-mediated disposal; ↑ triglyceride–glucose index	Marked metabolic inflexibility under placental insulin resistance	Persistent insulin resistance; type 2 diabetes and MASLD risk
Placenta & endothelium	↑ glycosylated fibronectin; early endothelial dysfunction	↑ sFlt-1/PlGF; glycocalyx injury; pre-eclampsia overlap	Vascular “memory”; accelerated cardiovascular risk
Pancreatic beta-cell	Adaptive expansion to meet demand	Failing expansion; stress and dedifferentiation in susceptible women	Reduced reserve; earlier progression to type 2 diabetes
Kidney	Hyperfiltration; subtle early strain	Albuminuria in some; shared angiogenic stress	Later albuminuria and chronic kidney disease
Heart & vasculature	↑ arterial stiffness; ↓ nitric-oxide bioavailability	Increased afterload; subclinical dysfunction	Hypertension and cardiovascular events
Lung (least established)	Low-grade inflammation; possible microvascular change	Possible altered ventilatory reserve; peri-partum respiratory risk	Hypothesis-generating; needs dedicated study

Entries are stage- and organ-specific; lung, MASLD and dedifferentiation entries are extrapolated from type 2 diabetes/CKM literature and flagged as hypothesis-generating. GLUT4, glucose transporter type 4; MASLD, metabolic dysfunction-associated steatotic liver disease; PlGF, placental growth factor; sFlt-1, soluble fms-like tyrosine kinase-1.↑, increased or higher than reference; ↓, decreased or lower than reference.

Several nodes of the model remain clearly speculative and are flagged as such: pulmonary involvement (low-grade inflammation, possible pulmonary-microvascular change and altered ventilatory reserve), postpartum MASLD risk in Southeast Asian GDM specifically, and beta-cell dedifferentiation markers (transcription-factor signatures, microRNAs) in human pregnancy. Each is inferred largely from the type 2 diabetes and general CKM literature rather than from GDM-specific longitudinal studies, and should be read as biologically plausible but hypothesis-generating, awaiting direct confirmation in GDM cohorts.

The unifying insight is molecular. The pathways that injure the endothelium and beta cell in GDM— mitochondrial reactive-oxygen-species generation, advanced glycation, nuclear factor-κB-driven inflammation, eNOS uncoupling, and beta-cell stress and dedifferentiation—are the same processes that drive CKM disease in non-pregnant adults (4, 47). Recognising GDM as a possible early clinical expression of this shared biology reframes antenatal care: pregnancy becomes the index event in a lifelong CKM-prevention trajectory rather than an isolated episode, and interventions targeting oxidative stress, inflammation and endothelial function acquire a plausible dual rationale—improving the index pregnancy and potentially modifying the mother’s long-term CKM course.

## 7 Precision diagnosis: the heterogeneity and subtypes of GDM

Treating GDM as a single entity obscures its biology. Physiological studies show that women meeting GDM criteria fall into distinct pathophysiological subtypes: a predominantly insulin-resistant subtype (higher body-mass index, marked insulin resistance), a predominantly insulin-secretion-deficient subtype (leaner, impaired beta-cell response), and a mixed subtype (31). These differ in ways that matter clinically—the insulin-resistant subtype carries the highest risk of large-for-gestational-age infants and adverse outcomes, and the subtypes track different maternal and offspring metabolic futures (32, 33). Analyses from the HAPO follow-up confirm that GDM subtypes are differentially associated with newborn and childhood metabolic outcomes, arguing that subtype, not merely the presence of GDM, should inform prognosis (34). Clustering studies show these differences are already detectable in early pregnancy (35). This heterogeneity is the rationale for the ADA/EASD Precision Medicine in Diabetes Initiative, which frames GDM as a condition to be stratified by phenotype, biomarker, and ultimately genotype (36, 37) (**Table 4**). The point is sharpest for lean Southeast Asian women, over-represented in the insulin-deficient subtype, in whom obesity-anchored screening and management may not fit the underlying physiology. In Tier 1–2 settings, phenotyping can nonetheless begin with simple, routinely available data (body-mass index, fasting glucose, basic lipids) before advanced biomarkers are considered. It is important to note that the subtype scheme itself was developed chiefly in non-Asian populations and requires validation in Southeast Asian cohorts before it can be treated as a regional standard.

**Table 4:** Pathophysiological subtypes of gestational diabetes and their clinical implications.

Subtype	Predominant defect	Typical features / outcomes	Management implication	Approach in resource-limited settings
Insulin-resistant	Severe insulin resistance	Higher BMI; greatest risk of large-for-gestational-age	Insulin sensitisation (lifestyle, metformin)	Lifestyle first; cautious metformin; monitor foetal growth
Insulin-deficient	Impaired insulin secretion	Leaner; common in Asian women; beta-cell limitation	May require earlier insulin	Lower threshold for early insulin; avoid over-reliance on metformin
Mixed	Both defects	Intermediate features	Individualised, often intensified	Individualised; insulin often needed; close monitoring

BMI, body-mass index. Subtypes are defined by indices of insulin sensitivity and secretion derived from the diagnostic OGTT, and are largely derived from non-Asian cohorts.

Subtype thinking can be extended into a multidimensional, multi-organ precision map (**Table 5**). GDM is not confined to the beta cell: skeletal muscle and liver supply the background insulin resistance; the placenta and vascular bed express early stress through glycosylated fibronectin, the sFlt-1/PlGF axis and arterial stiffness; and the beta cell mounts an adaptive expansion that may fail—with metabolic stress and dedifferentiation towards a progenitor-like, less insulin-secretory state under gluco- and lipotoxicity—in susceptible lean women. Characterising each woman along glycaemic (fasting- versus post-load-predominant), beta-cell (deficient versus preserved) and vascular–angiogenic axes, and identifying her dominant organ axis, yields a richer, testable phenotype than a single glucose value. The scheme is deliberately hypothesis-generating and awaits prospective validation, but its first axis needs only routine glucose testing, so it is implementable in tiered form even where advanced assays are unavailable.

**Table 5:** A multidimensional, multi-organ precision map of gestational diabetes.

Dominant axis / organ	Predominant defect	First-trimester markers	Pregnancy and CKM-prevention focus
Muscle–liver (insulin-resistant)	Skeletal-muscle and hepatic insulin resistance	↑ fasting glucose, ↑ triglyceride–glucose index, ↓ adiponectin	Lifestyle first, cautious metformin; postpartum weight, lipid and blood-pressure control, MASLD assessment
Placenta–vascular	Endothelial and angiogenic stress	↑ glycosylated fibronectin, ↑ sFlt-1/PlGF, ↑ arterial stiffness	Closer antenatal and pre-eclampsia surveillance; earlier postpartum cardiovascular and kidney follow-up
Beta-cell	Impaired secretion/dedifferentiation	Low OGTT-derived secretion indices; low C-peptide relative to demand; lean phenotype	Lower threshold for early insulin; postpartum beta-cell preservation, avoid prolonged glucotoxicity

CKM, cardiovascular–kidney–metabolic; MASLD, metabolic dysfunction-associated steatotic liver disease; OGTT, oral glucose tolerance test. Axes overlap; most women show a dominant but not exclusive pattern.↑, increased or higher than reference; ↓, decreased or lower than reference.

### 7.1 Beta-cell mass, stress and dedifferentiation

The beta-cell axis deserves separate emphasis, because it is where the lean Southeast Asian phenotype most clearly diverges. In healthy pregnancy, maternal beta-cell mass and function expand to meet the insulin demand imposed by placental hormones. GDM represents a failure of this adaptation. In many South and Southeast Asian women the defect is less one of insulin resistance than of limited secretory reserve: the beta cell cannot expand sufficiently, and under sustained gluco- and lipotoxicity it may experience metabolic stress and dedifferentiate, reverting towards a progenitor-like, less insulin-secretory state rather than dying outright. This biology may help explain why leaner women develop GDM at low body-mass thresholds and progress to type 2 diabetes disproportionately after pregnancy. Clinically, beta-cell stress can be approximated with tools already in use—OGTT-derived secretion indices, C-peptide relative to glucose, and early-postpartum fasting insulin and glucose—and women in the insulin-deficient, high-beta-cell-stress group may warrant a lower threshold for early insulin and structured, beta-cell-preserving follow-up. Whether circulating markers of dedifferentiation can one day identify these women prospectively is an open and important question, and human-pregnancy data on such markers remain sparse.

## 8 Precision treatment: individualising management

That treating GDM improves outcomes is well established: the ACHOIS and Landon trials showed that treatment of even mild GDM reduces serious perinatal morbidity, macrosomia, pre-eclampsia, and shoulder dystocia (38, 39). Management remains stepwise—medical nutrition therapy and physical activity first, pharmacotherapy when glycaemic targets are not met—but the choice and timing of pharmacotherapy is where precision is emerging. Insulin is the reference standard and crosses the placenta minimally. Metformin achieves comparable glycaemic control with less maternal weight gain and hypoglycaemia in the MiG trial, although many women still require supplemental insulin, and metformin crosses the placenta, keeping questions about long-term offspring effects open (40); in pregnancies complicated by type 2 diabetes, the MiTy trial showed that adding metformin to insulin improved some outcomes whilst reducing small-for-gestational-age risk (41). Metformin’s low cost and oral administration make it attractive across Southeast Asia, but the risk of foetal growth restriction in already lean, growth-restriction-prone populations warrants caution.

The precision-medicine agenda asks a sharper question: which woman should receive which treatment? A systematic review for the Precision Medicine in Diabetes Initiative found that treatment response in GDM varies with maternal phenotype—baseline glycaemia, body mass index, and markers of insulin resistance versus deficiency (42). Continuous glucose monitoring, earlier treatment in confirmed early GDM (15), and phenotype-guided drug selection together point towards management matched to subtype rather than applied uniformly. That future is not yet fully evidence-based, but the direction—from one-size-fits-all to stratified, phenotype-guided GDM care—is clear, and it depends on exactly the early, biomarker-rich characterisation described here.

Mapping therapy to the axes of **Table 5** generates concrete, falsifiable hypotheses. In insulin-resistant, vascular-high-risk women (raised triglyceride–glucose index, low adiponectin, early arterial stiffness), intensive lifestyle intervention and cautious metformin in pregnancy could be paired with assertive postpartum CKM care—lipid and blood-pressure control, kidney surveillance, and, after pregnancy and breastfeeding, where indicated under standard non-pregnancy CKM guidelines, GLP-1 receptor agonists or SGLT2 inhibitors selected by cardiometabolic and kidney-risk phenotype, with timing governed by lactation status and reproductive plans, per prevailing guidelines. In lean, insulin-deficient women with relatively preserved vascular markers, earlier physiological insulin and structured beta-cell-preserving follow-up may fit better. None of these pairings is yet proven in trials, but each is a testable direction for precision GDM care that a developing-country service could begin to evaluate prospectively. This diagnostic-to-treatment logic can be read at a glance (**Figure 4**).

**Figure 4:**
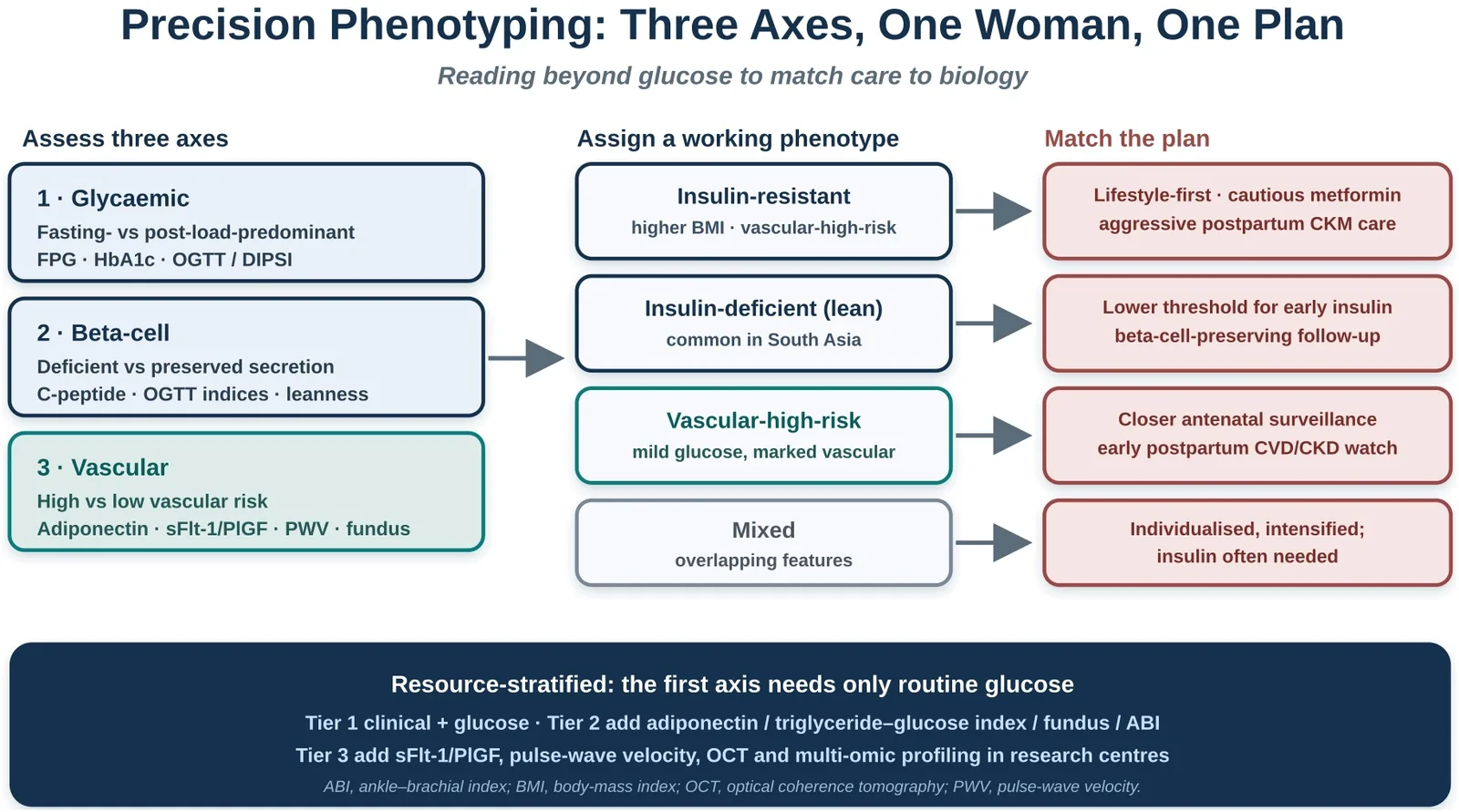
Precision phenotyping—from three axes to a plan. Each woman is characterised along glycaemic, beta-cell and vascular–angiogenic axes, assigned a working phenotype (insulin-resistant/vascular-high-risk, lean insulin-deficient, vascular-high-risk, or mixed), and matched to a pregnancy and postpartum CKM plan. Because the first axis needs only routine glucose, the scheme is resource-stratified across Tiers 1–3. Conceptual, hypothesis-generating schematic integrating existing evidence; not yet prospectively validated.

## 9 Gestational diabetes in Southeast Asia

Southeast Asia concentrates every argument in this review. Where recommendations here draw on non-Asian or multi-ethnic data, we label them explicitly as extrapolated and hypothesis-generating for Southeast Asia. Reported GDM prevalence is high and heterogeneous—of the order of 8% in Malaysia and Myanmar, 14% in Singapore, 17% in Vietnam, and up to 29% in Thailand—with much of the spread attributable to differing diagnostic criteria and to whether screening is universal or selective, rather than to biology alone (**Table 6**) (2, 43). Countries and institutions across the region variously apply IADPSG, WHO, NICE, Carpenter–Coustan, and older National Diabetes Data Group criteria, so a woman classified as having GDM in one setting might not be in another. Against constrained antenatal budgets, the universal-versus-selective screening question is not academic; universal 75 g testing detects more disease but strains services, and a validated, simple, non-fasting first-trimester test could be particularly valuable (23). Although this review centres Southeast Asia, the same realities—diagnostic heterogeneity, a lean insulin-deficient phenotype, and constrained antenatal resources—are shared across India and much of the developing world; DIPSI’s single non-fasting 75 g test is pragmatic but may miss fasting-predominant hyperglycaemia, and a first-trimester biomarker layer could reasonably be added on top of it (46).

**Table 6:** Reported gestational diabetes prevalence in selected Southeast Asian countries.

Country	Prevalence (approx.)	Criteria / screening	Note
Thailand	~29% (amongst the highest reported)	Carpenter–Coustan/IADPSG; often universal	Varies markedly with criteria used
Vietnam	~17%	IADPSG/WHO; increasingly universal	Rising with urbanisation and nutrition transition
Singapore	~11–14% (multi-ethnic)	IADPSG; universal in GUSTO	Ethnic variation (Malay, Indian, Chinese)
Malaysia	~8%	WHO/NICE; often selective	Selective vs universal screening differences
Myanmar	~8%	WHO; selective/limited	Limited data; likely under-ascertained

Estimates are indicative and not directly comparable because diagnostic criteria and screening strategies (universal vs selective) differ across studies and countries.

The regional phenotype compounds the diagnostic problem. Southeast Asian women develop GDM at lower body-mass thresholds than European-ancestry women and are over-represented in the leaner, insulin-secretion-limited subtype, so obesity-anchored risk-factor screening misses many affected women (2). Genetic susceptibility loci shared with type 2 diabetes—including MTNR1B, CDKAL1, TCF7L2, IGF2BP2, and GCKR, with adiponectin (ADIPOQ) variants—are prevalent in these populations (44), and ethnic differences in adiponectin and insulin-resistance patterns influence biomarker performance. Multi-ethnic Asian birth cohorts such as GUSTO in Singapore have been instrumental in dissecting these ethnic and dietary contributions and in developing population-appropriate prediction tools (45). First-trimester, biomarker- and vascular-based, subtype-aware detection is therefore not merely convenient for Southeast Asia but potentially corrective of the systematic under-detection produced by importing Western, obesity-centred models.

It is important, however, to separate what is established in Southeast Asian populations from what is extrapolated to them. Generated in South/Southeast Asian cohorts: the high and variable regional prevalence; the lean phenotype at lower BMI, spanning both insulin-resistant and insulin-secretion-limited subtypes; the pragmatic performance of DIPSI; and Indian triglyceride–glucose-index data, with the GUSTO cohort providing Asian first-trimester data. Extrapolated to the region and therefore requiring local validation: the TOBOGM early-treatment benefit (an Australian trial); HAPO and its follow-up; most first-trimester biomarker thresholds; the GDM-subtype scheme; and the ADA/EASD precision-medicine consensus, all derived chiefly from Western or multiethnic populations. Recommendations resting on the latter are offered here as hypotheses to be tested in Southeast Asian cohorts rather than as validated regional standards.

## 10 Practical implications for clinicians in developing countries

Translating these ideas into everyday antenatal practice, especially in resource-limited settings, can follow a pragmatic approach matched to what a centre can afford (**Table 7**; **Box 3**). A feasible Tier 1 pathway would offer every woman simple first-trimester risk stratification—age, body-mass index, family history of diabetes, previous GDM, and polycystic ovary syndrome—with readily available laboratory markers (fasting glucose, HbA1c, and a lipid profile). Where feasible, adding adiponectin or the triglyceride–glucose index refines early risk, and high-risk women should be flagged for structured postpartum CKM follow-up rather than pregnancy-limited care. In centres using DIPSI-only screening, piloting a combined early non-fasting biomarker step followed by a later OGTT, with prospective data capture, would build the local evidence the region lacks.

**Table 7:** Proposed tiered, resource-stratified strategy for early GDM risk assessment.

Tier	Setting	Assessment
Tier 1	Any antenatal clinic	Clinical risk factors (age, BMI, family history, prior GDM, PCOS) plus fasting glucose/HbA1c; DIPSI or OGTT per local protocol
Tier 2	Better-resourced hospitals	Add adiponectin, lipid profile and triglyceride–glucose index for refined early risk; flag high-risk women for postpartum CKM follow-up
Tier 3	Research/tertiary centres	Add advanced vascular, placental and evolving multi-omic and machine-learning profiling (e.g., sFlt-1/PlGF, arterial stiffness) for validation and discovery

BMI, body-mass index; CKM, cardiovascular–kidney–metabolic; DIPSI, Diabetes in Pregnancy Study Group India; OGTT, oral glucose tolerance test; PCOS, polycystic ovary syndrome.

Box 3 **Practical implications—for the clinic.**

In brief, a diagnosis of GDM should be treated as the opening of a lifelong cardiovascular–kidney–metabolic window rather than the end of a pregnancy problem: screen early and pragmatically, match therapy to phenotype, and—above all—flag every woman with GDM for structured postpartum follow-up (a 6-week OGTT, then annual glucose, blood-pressure, kidney and cardiovascular-risk review with periodic MASLD assessment). The seven-step checklist in **Box 4** operationalises this.

Box 4 **A seven-step implementation checklist.**

1. At the first antenatal visit, record a simple vascular baseline—blood pressure, and, where locally available and supported by trained interpretation, an adjunctive vascular reading (fundus imaging, nailfold capillaroscopy, ankle–brachial index or pulse-wave velocity). One clearly abnormal reading reclassifies the woman as vascular-high-risk.
2. Screen early and pragmatically: clinical risk factors with fasting glucose or HbA1c (or DIPSI where that is the local test), adding adiponectin or the triglyceride–glucose index if affordable.
3. Assign a working phenotype—insulin-resistant, insulin-deficient/lean, or vascular-high-risk—and let it set the intensity of care.
4. Match treatment: lifestyle-first with cautious metformin in the insulin-resistant; a lower threshold for early insulin in the lean, insulin-deficient; closer surveillance in the vascular/placental-high-risk.
5. Prescribe a pragmatic lifestyle package: whole grains and pulses over refined carbohydrate, portion control, and a 10–15-min post-meal walk—for the whole household.
6. Flag every woman with GDM in the record for lifelong CKM follow-up: a 4–12-week (typically 6-week) postpartum 75-g OGTT, then annual glucose, blood-pressure, kidney (eGFR/ACR) and cardiovascular-risk review, with MASLD assessment.
7. Continue care beyond delivery—provide the woman and her family a written prevention plan and a recall date. The whole pathway, from the first antenatal visit to lifelong prevention, is summarised in **Figure 5**.

**Figure 5:**
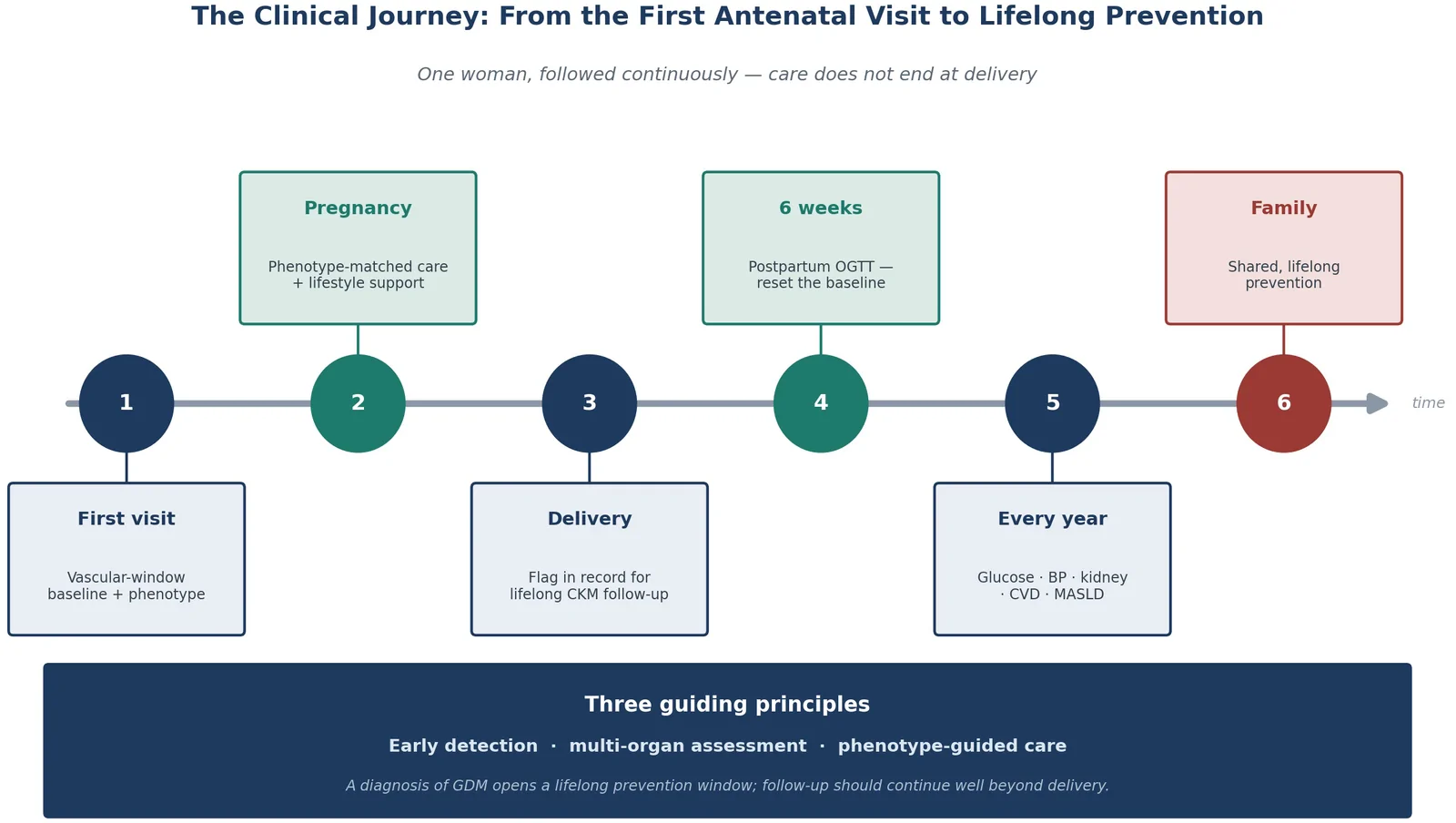
The clinical journey, from the first antenatal visit to lifelong prevention. A woman with GDM is followed as one continuous path: first-visit phenotyping and a vascular baseline, phenotype-matched pregnancy care with lifestyle support, a delivery-time flag for CKM follow-up, a 6-week postpartum reassessment, annual CKM review, and shared family prevention. Three guiding principles — early detection, multi-organ assessment, and phenotype-guided care — summarise the approach.

Feasibility, cost and infrastructure must be stated plainly. Tier 1 (clinical risk factors, blood pressure, fasting glucose or HbA1c, DIPSI) is realistic for essentially every primary antenatal clinic at negligible marginal cost. Tier 2 (adiponectin, triglyceride–glucose index, lipid profile, point-of-care glycosylated fibronectin) is realistic for a district hospital with basic laboratory or lateral-flow capacity, at a modest per-test cost, though assay standardisation, cold-chain and staff training remain barriers; adiponectin and lipids are realistic at the district level, whereas sFlt-1/PlGF and arterial-stiffness devices are not. Tier 3 (sFlt-1/PlGF, arterial-stiffness devices, multi-omic panels) is realistic only for tertiary centres with equipment, trained operators and quality assurance, and risks widening equity gaps if promoted for routine use. The model is designed to be entered at whatever tier a service can sustain.

Subtype-aware management can be applied even without advanced tools: leaner, insulin-secretion-limited women more often require earlier insulin, whereas heavier, insulin-resistant women may benefit most from intensified lifestyle measures and cautious metformin, mindful of foetal-growth considerations. Embedding this within a hospital system is largely organisational: a short structured antenatal proforma to capture early risk markers, and a simple electronic or register-based flag tagging every woman with GDM for lifelong CKM screening—annual dysglycaemia testing, blood-pressure and kidney assessment, and cardiovascular risk estimation—turns a pregnancy diagnosis into a durable prevention opportunity. Because offspring inherit both the metabolic exposure and often the household environment, the highest-risk phenotypes may be appropriate candidates for family-based lifestyle and CKM-risk programmes. The approach is operationalised as a seven-step checklist (**Box 4**; **Table 8**), and the woman's continuous care pathway is summarised in **Figure 5**.

**Table 8:** Implementation notes assigning a working phenotype in Tier 1–2 settings.

Phenotype	Minimal data to assign (tier 1–2)	Example profile	Suggested action
Insulin-resistant	BMI/waist, fasting glucose or HbA1c, triglyceride–glucose index (± adiponectin)	Higher BMI, high TyG index, family history	Lifestyle-first; cautious metformin; standard surveillance
Insulin-deficient/lean	Low/normal BMI, elevated glucose with low TyG index (± C-peptide)	Lean, early hyperglycaemia, modest insulin resistance	Lower threshold for early insulin; monitor foetal growth
Vascular/placental high-risk	BP plus one vascular reading (fundus/ABI/PWV) and/or glycosylated fibronectin or sFlt-1/PlGF	Any clearly abnormal vascular or placental marker	Closer antenatal surveillance; early postpartum CKM review

Phenotype is provisional and revisited at booking, at 24–28 weeks, and postpartum; simple proxies suffice in Tier 1–2. ABI, ankle–brachial index; BP, blood pressure; PWV, pulse-wave velocity; TyG, triglyceride–glucose.

### 10.1 A bedside vascular and organ-function toolkit

A recurring objection to precision, vascular-based care is that it needs technology the average South Asian clinic lacks. In fact, much of the vascular phenotype can be read with simple, inexpensive, repeatable bedside tests already present in—or easily added to—regional practice (**Table 9**). Their rationale is that GDM involves demonstrable early endothelial and arterial change, and each instrument provides a low-cost, non-invasive readout of that vascular phenotype, converting an abstract construct into an objective, chartable number that can be repeated to track response. On current evidence, the ankle–brachial index and pulse-wave velocity are the best supported as adjunctive risk markers and are reasonable for selective use in higher-risk women; fundus assessment is widely feasible but of adjunctive value; retinal optical coherence tomography-angiography and nailfold capillaroscopy remain exploratory in GDM. Existing studies link arterial stiffness and ankle–brachial-index abnormalities in pregnancy to broader cardio-renal risk; their GDM-specific screening utility, however, remains to be demonstrated. We emphasise that none of these is a validated screening test for GDM; they are offered as adjuncts that may flag a vascular-high-risk phenotype, recommended for selective rather than universal use, and as hypotheses for prospective evaluation (**Table 10** sets out the evidence status of each component of the model). Worked examples make them action-oriented: an ankle–brachial index <0.9 or a pulse-wave velocity clearly above the age-expected range reclassifies a woman as vascular-high-risk and triggers intensified postpartum CKM follow-up with tighter blood-pressure and lipid management; a clearly abnormal fundus or nailfold pattern similarly moves a woman from standard to high-risk surveillance. An abnormal reading should be interpreted in clinical context and, where appropriate, confirmed before it is used to intensify surveillance or trigger referral. Which components of this framework rest on established evidence, and which remain hypothesis-driven, are set out in.

**Table 9:** A bedside vascular and organ-function toolkit for GDM in resource-limited settings.

Test	Organ/axis probed	Availability & cost	Recommended use	Clinical trigger
Fundus imaging	Retinal microvasculature	Widely available; smartphone cameras low-cost	Adjunct (widely feasible)	Abnormal → tighter glucose/BP control, closer CKM follow-up
OCT/OCT-angiography	Retinal capillary density; FAZ	Tertiary/urban; growing	Exploratory (Tier 3)	Flags vascular-high-risk phenotype for surveillance
Nailfold capillaroscopy	Peripheral microcirculation	Very low cost (dermatoscope/USB microscope)	Exploratory	Abnormal → intensify surveillance
Ankle–brachial index	Macrovascular/peripheral arterial disease	Handheld Doppler + cuff; low cost	Best-supported adjunct; selective use	<0.9 → confirm and assess clinically for peripheral arterial disease; consider cardiovascular-risk evaluation and intensified CKM follow-up per local pathways
Pulse-wave velocity	Central arterial stiffness	Oscillometric device; moderate cost	Best-supported adjunct; selective use	High value → vascular-high-risk phenotype
Spirometry	Lung — ventilatory reserve	Low cost; widely available	Research (hypothesis-generating)	Peri-partum respiratory-risk flag

All are non-invasive, repeatable, and require no reference laboratory. BP, blood pressure; CKM, cardiovascular–kidney–metabolic; FAZ, foveal avascular zone; OCT, optical coherence tomography.

**Table 10:** Evidence-supported versus hypothesis-driven components of the CKM–GDM multi-organ precision model.

Component	Evidence status	Population(s)	Clinical implication	Research need
GDM → later T2D/CVD/CKD	Strong (longitudinal)	Global + some Asian	Justifies lifelong CKM follow-up	Regional long-term cohorts
Continuous glucose–risk (HAPO)	Strong	Multi-ethnic	Supports lower thresholds	Asian threshold calibration
Early (<20 wk) treatment benefit	Strong (1 RCT: TOBOGM)	Australian	Supports early screening	SE-Asian pragmatic trial
Lean insulin-resistant phenotype	Moderate–strong (regional)	South/SE Asian	Tailors phenotyping	Mechanistic + outcome studies
First-trimester biomarker prediction	Moderate	Mostly non-SE-Asian	Enables early triage	Prospective regional validation
GDM subtypes	Moderate	Chiefly non-Asian	Guides treatment matching	Outcome-linked validation
Vascular window as unifying signal	Hypothesis-generating	Association/mechanistic	Motivates vascular assessment	Prospective screening studies
Bedside vascular tools for GDM	Hypothesis-generating	Extrapolated from CVD	Adjunct risk flag	GDM-specific diagnostic studies
Phenotype-guided treatment benefit	Hypothesis-generating	None dedicated	Rationale for trials	Phenotype-stratified RCTs
ML early CKM-risk prediction	Emerging	Limited Asian	Potential triage	External validation, impact trials
Lung/MASLD/beta-cell dediff. nodes	Speculative	Extrapolated (T2D/CKM)	Hypothesis only	Dedicated GDM studies
CKM–GDM Multi-Organ Precision Model	Hypothesis-driven (this article)	Proposed	Organises care conceptually	Prospective validation

T2D, type 2 diabetes; CVD, cardiovascular disease; CKD, chronic kidney disease; RCT, randomised controlled trial; ML, machine learning; MASLD, metabolic dysfunction-associated steatotic liver disease.

### 10.2 Diet and exercise: a regional strategy

Optimising the benefit of early detection depends on what follows it, and here the regional context is specific. The typical North Indian diet is dense in refined carbohydrate—polished rice, refined-wheat roti, and sweets—with a high glycaemic load and relatively low fibre, superimposed on a lean but insulin-resistant, often physically inactive phenotype. A pragmatic strategy shifts carbohydrate quality rather than merely quantity: replacing part of the refined-wheat and white-rice intake with whole grains and traditional millets (bajra, jowar, ragi), increasing pulses (dal), vegetables and salad for fibre, moderating portions of rice and sweets, and spreading carbohydrate across smaller meals to blunt post-prandial peaks. Protein adequacy matters in largely vegetarian diets and is achievable with dal, curd, paneer and legumes. On the activity side, a 10–15-min walk after each major meal is a simple, culturally acceptable measure that lowers post-prandial glucose, supplemented by structured aerobic activity and resistance or yoga-based exercise to improve skeletal-muscle insulin sensitivity. These measures are low-cost, sustainable, and especially potent in the insulin-resistant, vascular-high-risk phenotype; because the household diet and activity pattern are shared, they also seed family-based prevention.

### 10.3 Implementation in routine practice

The seven-step checklist in Section 10.3 operationalises this (**Table 10**). The whole pathway, from the first antenatal visit to lifelong prevention, is summarised in **Figure 5**.

## 11 Towards integrated, early, and precise GDM care

**Table 8** sets out the minimal data needed to assign a working phenotype in Tier 1–2 settings.

These data converge on one programmatic idea: GDM care should be early, multi-organ, phenotype-guided, and explicitly embedded in CKM-prevention pathways in developing countries—and Southeast Asia is where it is most needed. Diagnosis should move earlier and become biomarker-informed rather than resting on a late, single glucose threshold; prediction should integrate metabolic markers (adiponectin, glycosylated fibronectin, and the triglyceride–glucose index) with vascular and angiogenic readouts (the sFlt-1/PlGF axis and, where feasible, arterial stiffness) in machine-learning models suited to local populations (4, 22, 23, 25); and both diagnosis and treatment should be stratified by pathophysiological subtype (31, 36, 37).

Machine-learning prediction, whilst promising, faces well-recognised obstacles that must be met before clinical use: models trained in one population frequently fail on external validation and generalise poorly across ancestries, BMI ranges, rural–urban settings, and laboratory platforms; small, imbalanced datasets with heterogeneous GDM labels invite overfitting; “black-box” models lack the explainability clinicians and regulators require; and few have been integrated into, or evaluated within, real antenatal workflows, including low-resource clinics without electronic records. Priorities are prospective external validation in Southeast Asian cohorts, calibration as well as discrimination, interpretable or explainable architectures, and prospective impact studies embedded in clinical pathways. Accordingly, we present machine-learning-based risk prediction explicitly as an emerging, hypothesis-generating direction rather than a ready-made clinical tool.

Concretely, three study designs would move the field, each mapped to a component of the model: a prospective multicentre first-trimester cohort across several Southeast Asian ancestries, measuring a fixed panel of metabolic, angiogenic, and vascular markers against later OGTT-defined GDM and offspring outcomes—validating the vascular window and first-trimester biomarker prediction; a pragmatic cluster-randomised trial testing whether a resource-stratified early-screening pathway (**Table 7**) improves outcomes versus usual care—validating feasibility of the tiered model and the phenotype-guided pairings; and a registered diagnostic-test-accuracy systematic review and meta-analysis of first-trimester vascular and angiogenic markers, prespecifying the lean Southeast Asian phenotype as a subgroup—validating and de-biasing the biomarker evidence. This last synthesis is the natural companion to the present review and is presented as a separate PROSPERO-registered protocol.

### 11.1 Strengths and limitations

Strengths of this review include the integration of diagnostic criteria, early prediction, the vascular mechanism, precision subtyping, and CKM consequences into one named framework; an explicit resource-stratified, developing-country, and Southeast Asian lens uncommon in existing syntheses; and a linked research agenda with a companion PROSPERO-registered protocol that moves the work beyond pure theory towards testable pathways. Its limitations follow from the same choices. It is narrative rather than systematic, so selection was purposive and no formal risk-of-bias or quantitative appraisal was performed. The biomarker and machine-learning literature is likely affected by publication bias, with positive diagnostic-performance studies overrepresented and negative or null findings underreported, so reported sensitivities and specificities probably overstate real-world performance. Persistent heterogeneity of diagnostic criteria (IADPSG, WHO, NICE, DIPSI, Carpenter–Coustan) means studies define GDM differently, limiting comparability and the pooling of prevalence and biomarker estimates. And several first-trimester biomarkers and the GDM-subtype framework, whilst promising, are not yet validated prospectively in Southeast Asian populations or embedded in outcome trials; the CKM–GDM models themselves are conceptual and require prospective validation before routine adoption. The registered diagnostic-test-accuracy synthesis is intended to address the bias and heterogeneity formally. These gaps define the agenda above rather than undercut the direction of travel.

## 12 Discussion

Gestational diabetes is being redefined on every axis at once: from a single contested glucose threshold towards biomarker-informed, early diagnosis; from a late-pregnancy disorder towards a first-trimester, vascular condition; from a transient complication towards a possible lifelong window into CKM disease; and from one-size-fits-all treatment towards subtype- and phenotype-guided precision care. Endothelial and arterial dysfunction may supply both the earliest signals and the mechanism that ties these axes together. For Southeast Asia, where the phenotype is distinctive, the prevalence high and variable, and antenatal resources constrained, moving prevention upstream—earlier, more mechanistic, and more precise and fitted to the region’s own physiology and health systems—is an attractive but as-yet-unproven direction.

The hypothesis is supported at differing levels of strength, which we flag explicitly to avoid over-interpretation (**Table 9**). Strong: the association of GDM with later type 2 diabetes, cardiovascular, and kidney disease; the early endothelial/vascular signal; and the benefit of treating early hyperglycaemia (TOBOGM). Moderate: the performance of first-trimester biomarker panels and machine-learning signatures. Weak/hypothesis-generating: the full multi-organ map (including lung involvement) and the phenotype-to-treatment pairings. Two interpretive cautions follow. First, predictive performance in the AUC 0.75–0.80 range identifies risk but is not equivalent to trial-level evidence that acting on it improves outcomes. Second, most biomarker data are associative; whether modifying biomarker-linked pathways changes maternal or offspring outcomes is unproven. The evidence strongly justifies viewing GDM as a CKM signal and instituting structured postpartum follow-up but does not yet confirm the benefit of complex precision-phenotyping algorithms, particularly in low- and middle-income settings.

We therefore emphasise that the CKM–GDM Multi-Organ Precision Model, the vascular window, and precision phenotyping are conceptual, testable frameworks, not validated clinical tools. Their components—early biomarker prediction, vascular phenotyping, subtype-guided treatment, and integrated CKM follow-up—should be prospectively validated in diverse South and Southeast Asian cohorts, ideally within the cohort and trial designs outlined above, before being incorporated into routine clinical practice, and any implementation should proceed stepwise, aligned with available infrastructure and accompanied by robust outcomes evaluation. Reframing GDM as an early cardiovascular–kidney–metabolic event nonetheless changes how we might counsel patients, design antenatal pathways, and plan lifelong follow-up—offering, if the framework proves out, a rare opportunity to move prevention decades upstream.

## Data Availability

No original data were generated or analysed for this narrative review; all data discussed are drawn from the cited published sources. Further enquiries can be directed to the corresponding author.
